# Hemorrhage promotes inflammation and myocardial damage following acute myocardial infarction

**DOI:** 10.1186/1532-429X-16-S1-O72

**Published:** 2014-01-16

**Authors:** Mihaela Pop, Xiuling Qi, Jennifer Barry, Bradley H Strauss, Graham A Wright, Nilesh R Ghugre

**Affiliations:** 1 Physical Sciences Platform, Sunnybrook Research Institute, Toronto, Ontario, Canada; 2 Department of Medical Biophysics, University of Toronto, Toronto, Ontario, Canada

## Background

Myocardial hemorrhage, in association with microvascular obstruction (MVO), has recently been speculated to be a new independent predictor of adverse outcomes following acute myocardial infarction (AMI) [1,2]. However, whether hemorrhage is simply a marker of severity or is a cause of myocardial damage and adverse remodeling post-AMI is still under question. Our previous study suggested that hemorrhage may contribute to MVO following reperfusion [3]. The purpose of this study was to mechanistically determine whether hemorrhage, per se, worsens infarct and MVO size and tissue inflammation following an ischemic insult.

## Methods

Myocardial hemorrhage was artificially induced in a porcine model via intracoronary injection of collagenase as previously described [3]. The study involved three groups of animals (N = 9) subjected to ischemia-reperfusion injury in the left anterior descending artery (LAD): Group 1 (N = 3): 8 min ischemia with collagenase; Group 2 (N = 3): 45 min occlusion with saline; and Group 3 (N = 3): 45 min occlusion with collagenase. Imaging was performed on a 3T MRI scanner (MR 750, GE Healthcare) at 24 hrs post-reperfusion. Edema/Inflammation was evaluated by T2 quantification using a T2-prepared spiral sequence and hemorrhage was identified by T2* determined using a multi-echo gradient-echo acquisition. Infarct and MVO size was computed using early and late enhancement imaging (EGE, LGE). Explanted hearts were sectioned and assessed by gross pathology.

## Results

**Figure 1:**
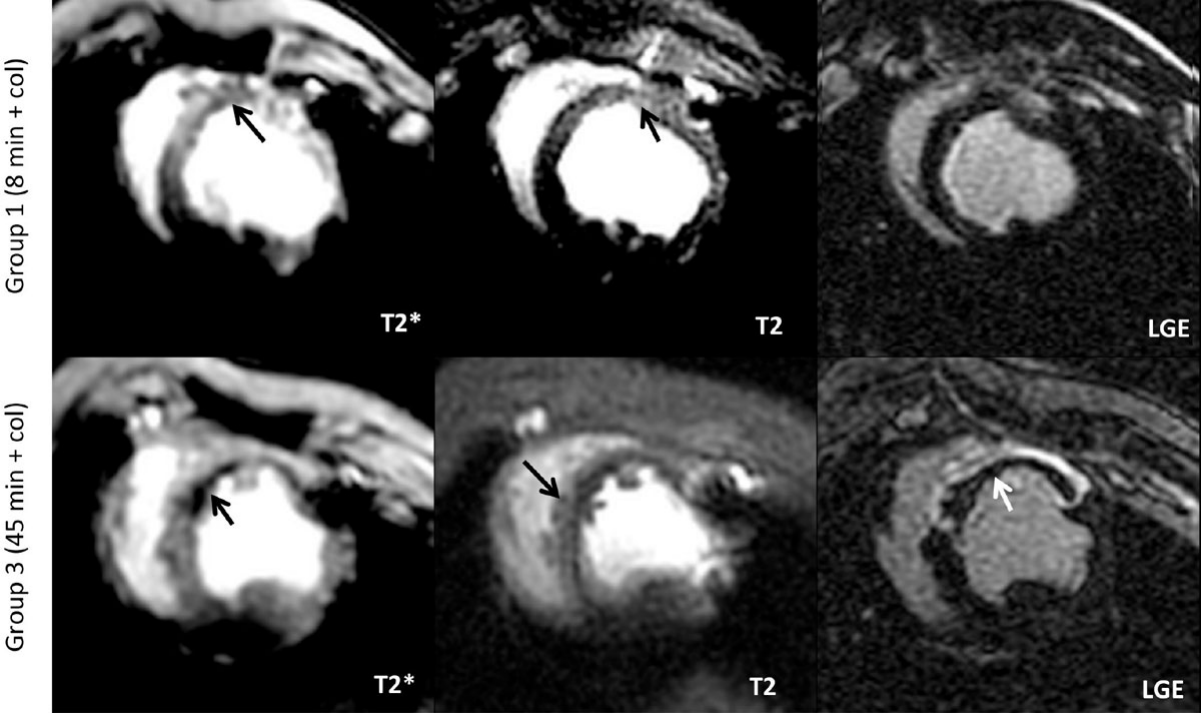
**Representative CMR images from animals in Groups 1 and 3**. T2*-weighted images (TE = 16 ms) demonstrate hemorrhage in both groups (arrows) but greater in Group 3. T2-weighted images (TE = 88 ms) identify elevated signal or edema (arrows) in the periphery of the hemorrhage or infarct core. Note that hemorrhage with greater ischemic insult in Group 3 is associated with a larger infarct size and the presence of MVO (arrow). Infarction was minimal in Group 1.

**Figure 2:**
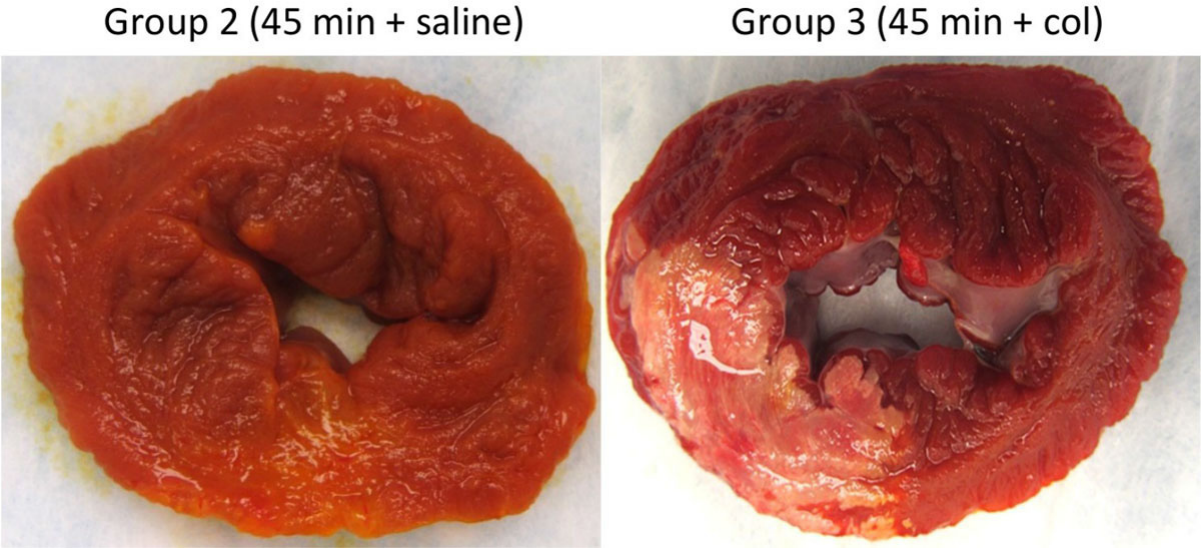
**Gross pathological sections of porcine hearts subjected to 45 min occlusion treated with either saline or collagenase (col)**. TTC staining indicates region on necrosis appearing white. Note that collagenase induced haemorrhage appears red on TTC stain and is associated with extended necrosis.

Group 1 demonstrated minimal infarction (only in 1 animal, 1.3 g) with significant hemorrhage as indicated by T2* (Figure 1) where as Group 2 was non-hemorrhagic with a small infarction (1.3 ± 0.8 g). In contrast, animals in Group 3 demonstrated greater hemorrhage (Figure 1), an infarct size significantly larger than the other two groups (10.7 ± 3.2 g, p = 0.03) and a higher incidence of MVO (2.5 ± 3.7 g). In Group 1, edema (measured by T2) near hemorrhagic sites was mild but detectable (43.8 ± 0.4 vs 36 ± 0.9 ms remote, p = 0.01) suggesting that hemorrhage itself is associated with an inflammatory response. In Group 2, edema in the infarcted tissue, was also mild (44.2 ± 1.1 vs 37.5 ± 0.7 ms remote, p < 0.01) where as it was extensive in Group 3 (51.8 ± 3.1 vs 37.9 ± 0.6 ms remote, p = 0.02). Edema severity in Group 3 was significantly greater than the other two groups (p < 0.05). TTC staining confirmed the CMR observations (Figure 2).

## Conclusions

Our novel findings demonstrate that hemorrhage is an important component of ischemia-reperfusion injury and may not simply be a bystander but an active contributor to cell damage and inflammation beyond the initial ischemic insult. A mechanistic understanding of the pathophysiology of reperfusion hemorrhage in vivo in the setting of AMI by CMR will potentially aid better management of the high-risk patients who are prone to adverse long-term outcomes.

## Funding

The studies were funded by the Heart and Stroke Foundation of Canada, Grant# 000334.
